# Bisphenol A and Its Analogues Deteriorate the Hormones Physiological Function of the Male Reproductive System: A Mini-Review

**DOI:** 10.3390/biomedicines9111744

**Published:** 2021-11-22

**Authors:** Asma’ ‘Afifah Shamhari, Zariyantey Abd Hamid, Siti Balkis Budin, Nurul Jehan Shamsudin, Izatus Shima Taib

**Affiliations:** 1 Centre for Diagnostic, Therapeutic and Investigative Studies, Faculty of Health Sciences, Universiti Kebangsaan Malaysia, Jalan Raja Muda Abdul Aziz, Kuala Lumpur 50300, Malaysia; p109998@siswa.ukm.edu.my (A.‘A.S.); zyantey@ukm.edu.my (Z.A.H.); balkis@ukm.edu.my (S.B.B.); 2 Centre for Toxicology and Health Risk Research, Faculty of Health Sciences, Universiti Kebangsaan Malaysia, Jalan Raja Muda Abdul Aziz, Kuala Lumpur 50300, Malaysia; nuruljehan@ukm.edu.my

**Keywords:** aromatase, bisphenol F, bisphenol AF, bisphenol S, HPG axis, steroidogenesis, spermatogenesis

## Abstract

BPA is identified as an endocrine-disrupting chemical that deteriorates the physiological function of the hormones of the male reproductive system. Bisphenol F (BPF), bisphenol S (BPS), and bisphenol AF (BPAF) are actively explored as substitutes for BPA and are known as BPA analogues in most manufacturing industries. These analogues may demonstrate the same adverse effects as BPA on the male reproductive system; however, toxicological data explaining the male reproductive hormones’ physiological functions are still limited. Hence, this mini-review discusses the effects of BPA and its analogues on the physiological functions of hormones in the male reproductive system, focusing on the hypothalamus-pituitary-gonad (HPG) axis, steroidogenesis, and spermatogenesis outcomes. The BPA analogues mainly show a similar negative effect on the hormones’ physiological functions, proven by alterations in the HPG axis and steroidogenesis via activation of the aromatase activity and reduction of spermatogenesis outcomes when compared to BPA in in vitro and in vivo studies. Human biomonitoring studies also provide significant adverse effects on the physiological functions of hormones in the male reproductive system. In conclusion, BPA and its analogues deteriorate the physiological functions of hormones in the male reproductive system as per in vitro, in vivo, and human biomonitoring studies.

## 1. Introduction

Bisphenol A (BPA) (*4,4′-dihydroxy-2,2-diphenyl propane*) is an organic synthetic monomer, reported to be one of the most produced chemicals in the world. About 7 million tonnes of BPA was produced in 2013, and this figure is estimated to be growing annually [1,2]. BPA was initially synthesized by the scientist Aleksandr Pavlovich Dianin in 1891. BPA’s resistance to high temperatures, shatterproof nature, and electrical insulation capability has led to this chemical being predominantly used in most epoxy resin and polycarbonate plastic industries [3,4,5]. Epoxy resin is a precursor chemical of any product that requires resilience and high durability, such as a dental sealant, amalgams, tanks, sports equipment, roads, structural adhesives, and coating materials in cans [6,7,8,9]. Polycarbonate plastic is also used as packaging or as containers for many daily consumable products, such as vegetables, fruits, milk bottles, plastic bags, and food and beverage containers. Furthermore, these polycarbonate plastics can also be involved in the packaging of personal care products such as shampoo, body wash, and cosmetics [7,8,9,10,11,12,13].

The extensive use of BPA in various industries has resulted in environmental pollution: BPA has reportedly been found in rivers, lakes, soils, sediments, home dust, and air [14,15,16,17,18]. The most alarming concern is that BPA has also been detected in human biological samples, such as in the blood, urine, sweat, breast milk, and in the umbilical cord [19,20,21]. BPA in human biological samples is expected, as it can be found in their food and drinking water, indicating that the most common source of BPA exposure in humans is oral [22,23,24,25]. Furthermore, BPA can also be exposed to humans via the dermis, which mostly occurs when someone is in indirect contact with thermal receipt paper. Humans may also be exposed to BPA through inhalation [26,27,28,29]. Recently, the negative impact of BPA has been widely reported by scientists and has been identified as a well-known endocrine-disrupting chemical (EDC) [30,31,32]. Previous studies have reported that exposure to BPA causes many diseases, such as tumours, cardiovascular diseases, neurodegenerative diseases, metabolic diseases, such as diabetes mellitus, autoimmune diseases, and male and female infertility, and can disturb the development of children, babies, and foetuses [29,33,34,35,36,37,38]. BPA increases the risk of transgenerational toxicity effects in the offspring, proven by an increase in epigenetic markers such as H3K9Ac and H3K27Ac in the spermatozoa due to parental BPA exposure [39,40].

The extensive adverse effects of BPA on human health have raised alarming awareness in society. Therefore, this chemical is being replaced with its analogues. Among the commonly used BPA analogues are bisphenol F (BPF), bisphenol S (BPS), and bisphenol AF (BPAF) [18,41,42,43,44]. These analogues share the same basic structure as BPA, where the two benzene rings are attached either with the short carbon or with other chemical chains [45,46]. However, these BPA analogues did not undergo proper toxicity evaluation testing before being used in the industry [47]. In 2015, the Environmental Protection Agency (EPA) reported a negative impact of BPA analogues on the aquatic system, environment, and human health [48]. Like BPA, its analogues also result in carcinogenicity, genotoxicity, neurotoxicity, reproductive toxicity, and developmental disorders [48]. Moreover, these analogues are also being categorized as EDCs.

Evidence showing that BPA and its analogues cause male infertility is growing. The most common mechanisms involved in toxicant-induced male infertility are oxidative stress and reproductive hormonal imbalance [49,50]. BPA and its analogues are also reported to exert oxidative stress in the plasma, testis, and sperm, resulting in spermatogenesis disturbance [51,52]. This disturbance leads to a reduction in sperm quality and testicular abnormalities, as shown by Leydig cell (LC), Sertoli cell (SC), and germ cell dysfunction [53]. BPA and its analogues also exhibited endocrine-disrupting activities in the male reproductive system, shown by the disturbance in the hypothalamus-pituitary-gonad (HPG) axis and steroidogenesis [54,55,56]. BPF, BPS, and BPAF share a property with oestradiol (E2), that is, these analogues possess the ability to activate the oestrogenic pathway in the human body system [57,58]. Their capability to mimic oestrogen-like properties causes a deterioration in the physiological function of the male reproductive system. Like other toxicants, BPA and its analogues display their affinity to bind to the oestrogen receptor (ER), such as ERα, ERβ, and oestrogen-related receptor gamma (ERR-γ) [56,59,60,61,62]. Furthermore, these chemicals also have the ability to disturb gene expression, such as Kiss1 and CYP19A1, which are involved in the HPG axis and steroidogenesis, respectively [63]. Recently, the effects of BPA and its analogues on these three significant pathways, in the form of the HPG axis [56,59,62,64,65,66,67,68,69,70,71,72,73,74,75,76,77], steroidogenesis [55,56,59,64,65,66,67,68,69,70,74,75,76,77,78,79], and spermatogenesis outcomes [64,65,66,67,68,69,70,75,76,77,80,81,82,83,84,85], have been well reported. To the best of our knowledge, the link between these three pathways in the exposures of BPA and its analogue is limited. Hence, this review was performed to connect the HPG axis, testicular steroidogenesis, and spermatogenesis outcomes after exposure to BPA and its analogues, leading to male reproductive toxicity

## 2. Bisphenols and Hypothalamus-Pituitary-Gonadal Axis in Male Reproductive System

The hypothalamus-pituitary axis is the main centre regulating endocrine hormone production in the human body, including the male reproductive system [2]. The hypothalamus releases the hormones responsible for stimulating the neuroendocrine activity of the pituitary glands, either in the anterior or posterior gland. One of the neuroendocrine activities regulated by the anterior pituitary gland is the HPG axis [3]. The HPG axis encounters three levels of hormone production: the hypothalamus releases the gonadotropin-releasing hormone (GnRH), the anterior pituitary gland secretes the follicle-stimulating hormone (FSH) and luteinizing hormone (LH), and the testis, specifically the LC, synthesises testosterone. GnRH, which is released from the hypothalamus, stimulates the anterior pituitary gland to release FSH and LH. Both hormones act on the testes to release target hormones, such as testosterone, oestrogen, progesterone, and inhibin. The primary purpose of this mechanism is to achieve homeostasis balance and modulate the positive and negative feedback of hormone regulation [4,5]. GnRH, LH, and FSH secretion are controlled by the neuropeptide kisspeptin (KiSS1), which is regulated by gene kiss1 [6,7,8]. Generally, the kiSS1 and G protein-coupled receptor 54 (GPR54) complex are involved in HPG axis feedback regulations [86]. KiSS1 binds to GPR54, also known as the kiss1 receptor, to form a Kiss1/GPR54 complex. This complex regulates the neuroendocrine reproductive axis by targeting the GnRH neuron to stimulate GnRH release [87]. Subsequently, GnRH stimulates neuron transmission at the anterior pituitary gland to induce the secretion of gonadotropic hormones, specifically LH and FSH. Furthermore, ERα also plays a significant role in regulating reproductive and sexual behaviour [88,89]. Once E2 binds to ERα in the hypothalamus, it suppresses GnRH secretion [90].

Several experimental studies revealed that BPA and its analogues, such as BPF, BPS, and BPAF, disturbed the HPG axis via KiSS1 and ERα by targeting mRNA gene expression. However, BPA and its analogues have either direct or indirect effects and are still controversial. Previous studies have reported that laboratory animals, such as rats and zebrafish, exposed to BPA showed an increase in Kiss1 mRNA expression in the brain [62,71,72,73]. Exposure to BPA at a dose of 50 µg/kg/bw via drinking water caused an increase in Kiss 1 mRNA expression [71]. The same findings were also recorded in the offspring and pups of rats and the transgenic embryo of zebrafish [62,72,73]. Exposure of zebrafish embryos to 1000 μg/L of BPA and BPS at 120 h post-fertilization (hpf) revealed an increased expression of the Kiss1 gene. Moreover, the Kiss 1 receptor was highly expressed, leading to an increased number of GnRH3 neurons in the hypothalamus [62]. GnRH3 neuron is a neuromodulator that indirectly controls the pituitary gonadal axis of the reproductive system in zebrafish [91,92]. Furthermore, Yang et al. [56] found that male zebrafish in an aquarium containing 0.1 and 1 mg/L BPF showed an increase in the expression of GnRH receptors (GnRHR1 and GnRHR2), which influences the increase of GnRH neurons. Another BPA analogue, BPAF, has also been reported to disturb the HPG axis in the offspring of male zebrafish by increasing the mRNA expression of gnrh2, fshβ, and lhβ [74]. In zebrafish, gnrh2, fshβ, and lhβ are orthologous to human GNRH2, FSHβ, and LHβ, respectively.

BPA and its analogues also showed the ability to interfere with ERα in in vivo and in vitro studies. The BPA and its analogues, BPF and BPAF, increased the binding affinity towards ERα, while BPS has no effect on this receptor in zebrafish embryos [59]. This observation is supported by in vivo studies where subcutaneous exposure to BPA at a dose of 50 mg/kg/bw for two days increased the expression of ERα in the hypothalamus [73]. The same finding was also shown in the transgenic zebrafish embryo exposed to BPA and BPS at doses of 1000 μg/L and 100 μg/L, respectively. In contrast, perinatal exposure to BPA at a dose of 50 µg/kg/bw via drinking water caused a decrease in the expression of both ERα and β in the hypothalamus of male Wistar rats during adulthood [71]. The increase of Kiss1 expression in the brain increases GnRH secretion, stimulating LH and FSH secretion. A study by Bai et al. [72] found that perinatal exposure to BPA at a dose of 2 µg/kg/bw increased the GnRH neuron in the brain, leading to an increase in LH levels in the blood of male rat offspring.

In contrast, several previous studies have reported a decrease in the FSH and LH levels in the blood of adult male rats when exposed to various dosages of BPA ranging from 25 mg/kg/bw to 200 mg/kg/bw either via oral gavage or intraperitoneal injection [64,65,66,67,75,76,77,80]. A similar finding was also shown in rats who were exposed to BPA analogues. A study by Ullah et al. [68] showed that BPF at a dose of 1 mg/kg/bw via oral gavage significantly reduced the LH and FSH levels in the plasma of male rats. Therefore, from the previous findings, we may assume that BPA and its analogues at a lower dosage may increase LH and FSH levels via Kiss1 expression. However, a contrasting finding was noted when BPA was exposed at a higher dosage. LH in the blood binds to the LH receptor on the LC membrane to stimulate testosterone synthesis [93]. A previous study reported a decrease in the testosterone levels in the blood of adult male rats when exposed to BPA either during adulthood or exposure of offspring via oral gavage, subcutaneously, or intraperitoneal injection [64,65,66,75,76,77,80].

Furthermore, exposure to BPF and BPS for 28 days in male rats also decreased testicular and plasma testosterone [52,68,69,70]. BPA and its analogues, BPF and BPAF, also caused a decrease in testosterone levels in the plasma or testis of male rats and zebrafish [42,72,74,75,76]. In contrast, a study by Stoker et al. [71] reported that perinatal exposure to BPA at a dose of 50 µg/kg/bw via drinking water caused an increase in the testosterone level of male Wistar rats during their adulthood. The contradictory results might be due to the dose, route, and length of BPA exposure. The effects of BPA and its analogues on the correlation between Kiss1, ERα, and GnRH in the brain with the levels of FSH, LH, and testosterone in the plasma is still doubtful. The differences may be due to the different doses, way of exposure, types of animals, and study duration. There might also be other mechanisms involving the correlation; for instance, the bisphenols can disrupt the mechanism of negative/positive feedback system in regulating the reproductive hormones or may do so via competitive inhibition at the receptor level either in the brain or the testis. Table 1 shows a summary of the effect of BPA and its analogues on the HPG axis involving the male reproductive system.

## 3. Bisphenols and Steroidogenesis

Testicular steroidogenesis is another important process in regulating the normal physiology of the male reproductive system. Steroidogenesis products such as testosterone, oestrogen, inhibin B, and progesterone play an essential role in maintaining the homeostasis of hormones in blood circulation. LC is a well-known site for steroidogenesis, particularly in the male reproductive system [94]. Steroidogenesis occurs in two different locations in the LC: the mitochondria and endoplasmic reticulum [93,94]. LH in the circulation binds to the LH receptor (LHR) at the LC membrane, thus activating the G protein groups to form the LHR/G protein complex. This complex activates two pathways by increasing cyclic adenosine monophosphate (cAMP) production and allowing the entry of arachidonic acid (AA) into the LC [95]. Next, cAMP activates protein kinase A (PKA) and mitogen-activated protein kinase (MAPK) for the stimulation of the steroidogenic acute regulatory (StAR) protein. This StAR protein is responsible for the transportation and movement of cholesterol from the outer membrane to the inner membrane of mitochondria [96,97].

Meanwhile, the presence of AA in the LC helps control testosterone production by inhibiting cholesterol movement to the mitochondria [98,99]. AA produces prostaglandin-E2 (PGE-2) via the activation of cyclooxygenase-2 (COX-2) and inhibits StAR functions [100,101,102]. Cholesterol is the primary substrate acting as a precursor in testicular steroidogenesis [94,96]. In mitochondria, cholesterol is converted to pregnenolone through the action of CYP11A1 [103]. The pregnenolone then moves into the endoplasmic reticulum, and the steroidogenic cascade of enzyme reaction that takes place involves the CYP450 enzyme (CYP17) and hydroxy steroid dehydrogenase (HSD) enzymes (3β-HSD and 17β-HSD). The conversion of pregnenolone into testosterone can be divided into two pathways (Δ4 and Δ5). These pathways can be alternated depending on the binding affinity of the CYP17 towards the substrates, 17α-hydroxy pregnenolone and 17α-hydroxyprogesterone, which activate the Δ5 and Δ4 pathways, respectively [103]. Humans mainly undertake this activity through the Δ5 pathway, while rats and mice mostly take the Δ4 pathway. In normal physiology, in testicular steroidogenesis, the Δ5 pathway is less prone to be converted to the alternative pathway synthesizing the E2 than the Δ4 pathway.

Nowadays, growing evidence has demonstrated the ability of bisphenols to disturb the steroidogenesis pathway. StAR, a protein responsible for transporting and moving cholesterol into the mitochondria, is among the proteins affected by exposure to BPA and its analogues. The gene and protein expression of StAR was decreased in adult male rats exposed to BPA for 28 days and 42 days, respectively, at a dose of 200 mg/kg by oral gavage [65,67]. Furthermore, BPA analogues such as BPF, BPAF, and BPS also caused a decrease in the expression of StAR mRNA. The expression of StAR mRNA was decreased in BPF and BPAF in the adults and offspring of male zebrafish, respectively [56,74]. Meanwhile, Eladak et al. [78] found that BPF and BPS also significantly decreased the expression of StAR mRNA in mouse foetal testicular cells (mFeTA) at a concentration of 10,000 nmol/L. The disturbance of the StAR mRNA expression may lead to the deterioration of testicular steroidogenesis due to the disturbance in cholesterol transportation and movement into the mitochondria in the LC.

Previous findings have also shown disturbance in the gene and protein expression of cytochrome P450 and HSD enzymes either in the mitochondrial or reticulum endoplasmic of LC, such as CYP11A1, CYP 17A1, 3β-HSD, and 17β-HSD. Exposure to BPA at a dose of 200 mg/kg for 28 days reduced the gene expression of CYP11A1 in the testicular mitochondria of male Sprague-Dawley rats [67]. Furthermore, exposure to BPA at the same dose for 42 days also decreased the protein expression of CYP11A1 [65]. In contrast, the expression of CYP11A1 was found to increase in adults and embryos of male zebrafish exposed to BPF and BPAF, respectively [56,74]. The disruption of CYP11A1 either involving the gene or protein expression decreases the conversion of cholesterol to pregnenolone in the mitochondria of LC. The CYP17A1 and 3β-HSD gene expression involved in the steroidogenic enzyme cascade in the endoplasmic reticulum also decreased in BPA-intoxicated rats [65]. The 17β-HSD, 3β-HSD, and CYP17A1 protein expression also decreased in the testis of male Sprague-Dawley rats exposed to BPA [80]. The same findings were also noted with exposure to BPA analogues in either in vivo or in vitro studies. BPF and BPAF exposure decreased CYP17 expression in the testis of zebrafish, while 17βHSD was found to be decreased in the testis of adult male zebrafish after 21 days of exposure to BPF [56,74]. An in vitro study conducted by Eladak et al. [78] found that BPF and BPS at the highest dose (10,000 nmol/L) caused a decrease in the expression of HSD3β1 and CYP17A1 in mFeTA after three days of exposure.

Furthermore, BPF and BPAF also decreased the gene expression of HSD3β2 and CYP17A1 in the human adrenocortical carcinoma cell line [79]. However, only BPS exposure has been reported to decrease the gene expression of CYP17A1 in the same cell line [79]. Hence, the interference of the steroidogenic enzymes, demonstrated by the changes in the gene and protein expression of 17β-HSD, 3β-HSD, and CYP17A1, may be the reason for the abnormal testosterone level in BPA, BPF, BPS, and BPAF exposure [56,65,74,78,79].

Most exposure to BPA and its analogues causes decreased testosterone levels due to the low level of LH and the disturbance of the steroidogenic enzyme cascade in the LC [52,56,64,65,66,67,68,69,72,74,75,76,77,78,79]. In contrast, Roelofs et al. [55] reported that exposure to BPF and BPS for 48 h caused an increase in the level of testosterone in the MA-10 LC culture. Moreover, Stoker et al. [71] also reported an increase in the testosterone level in male Wistar rats during their adulthood when exposed to BPA in their perinatal stage [71]. Decreasing testosterone synthesis activates the backdoor pathway, whereby dehydroepiandrosterone (DHEA) is converted into androstenedione (AD). AD is responsible for the Δ4 pathway, and this pathway poses a high risk of stimulating the overproduction of E2. This backdoor pathway is also known as the alternative pathway in testicular steroidogenesis involving the activation of p450 aromatase. The activation of aromatase is exhibited by the increased gene and protein expression of CYP19A1, which leads to the activation of cAMP. This mechanism causes the overproduction of E2 in the testis, which also presents as an effect of BPA and its analogues [56,59,72,74,75,76,79]. However, studies conducted by Stoker et al. [71] and Alboghobeish et al. [64] found a decrease in E2 levels in the blood circulation of adult male rats exposed to BPA. The disruption in this steroidogenesis pathway leads to a disturbance in sperm synthesis known as spermatogenesis. Table 2 shows the effects of BPA and its analogues on steroidogenesis in the male reproductive system.

## 4. Bisphenols and Spermatogenesis

Spermatogenesis occurs within the seminiferous tubules in the testis. The germ cells, such as spermatogonia, spermatocyte, and spermatid, undergo various stages of spermatogenesis to form sperm. Spermatogenesis occurs via specific processes, such as proliferation, differentiation, mitosis, meiosis, and spermiogenesis, to develop mature spermatozoa. Among these specific processes, proliferation, differentiation, and mitosis occur in the basement membrane, while the remaining processes occur in the adluminal compartment [104]. The blood–testis barrier (BTB) is formed after the basal membrane to protect the microenvironment of the adluminal compartment for the processes relevant to that area. Therefore, the germ cells found in the basement membrane are more vulnerable to any toxicants than the germ cells found in the adluminal compartment [93]. The integrity of the BTB is also crucial because changes in its structure may affect the production and morphological structure of the sperm [95]. Spermatogenesis involves not only different stages of germ cells but also SCs. These cells secrete pyruvate and lactate to nourish germ cells during their development and are responsible for the organization of the germ cells [103]. Therefore, any disturbance in the SC causes degeneration and disorganization of the germ cells. Testosterone plays a critical role in spermatogenesis owing to its ability for BTB maintenance, meiosis, Sertoli-spermatid adhesion, and the release of mature spermatozoa [104]. Testosterone maintains the remodelling of the BTB by binding with AR to form the protein involved in the integrity of tight junctions. Testosterone is needed in the completion of meiosis during the development of spermatocytes. Moreover, testosterone also plays an essential role in preventing the elongated spermatid from being released earlier. However, the testosterone hormone helps release the mature spermatozoa into the lumen of the seminiferous tubule, thus preventing spermatozoa from being engulfed by the SC [104].

BPA and its analogues were reported to disturb spermatogenesis by diminishing the BTB integrity, changing testicular histopathology, and causing sperm defects [52,64,65,66,67,68,69,70,75,76,77,80,81]. A study carried out by Li et al. [105] found a disturbance in the BTB of male Wistar rats, which was proven by the reduction of occludin and nectin-3 when exposed to BPA in a dose-dependent manner. The reduction of occludin was also found in the SCs, which were exposed to BPA in in vitro studies [82,83]. Furthermore, both studies also showed a decrease in the ZO-1 protein level [82,83]. The disturbances of these proteins lowered the integrity of BTB, which was proven by the reduction in cell viability and androgen receptor (AR) after 6 h of BPA exposure [82]. Moreover, Feng et al. [83] also found that the reduction of occludin and ZO-1 in SCs significantly perturb the tight junction barrier, lowering the integrity of the BTB. The disruption of BTB integrity may allow germ cells in the adluminal compartment to be exposed to toxicants, leading to the disturbance of spermatogenesis in the seminiferous tubules.

Spermatogenesis disruption after exposure to BPA and its analogues can be shown by histological observations, such as reduction in the diameter and epithelial height of germ cells, atrophy and separation of germinal epithelium, and irregular seminiferous tubule structure [66,67,80]. Previous studies reported that BPA exposure causes histopathological changes, proven by the vacuolation, degeneration, and disorganization of germ cells [66,67,80]. The vacuolation and degeneration of germ cells were reported after BPA exposure either via oral gavage for 52 days or intraperitoneal injection on alternate days for 30 days in adult male Sprague-Dawley rats [66,80]. The spermatogenesis process was found to be weak, arrested in the seminiferous tubule of adult male Wistar rats exposed to BPA at a dose of 50 mg/kg via oral gavage for 14 days [77]. Furthermore, the same study also found that spermatocytes are among the most affected germ cells in BPA-intoxicated rats [77]. Wang et al. [67] found that BPA at a dose of 200 mg/kg via oral gavage caused disorganization of germ cells [67]. However, these changes were not observed in BPA analogue-intoxicated rats. Moreover, BPA analogues such as BPF and BPS cause spermatids to become longer, and the absence of mature spermatozoa in the lumen of seminiferous tubules disrupts spermatogenesis in adult male Sprague-Dawley rats [65,68,69]. According to Liang et al. [81], BPA and its analogues (BPS and BPAF) decrease cell viability and increase the DNA damage of the spermatogonia cell line (C18-4). Among these bisphenols, BPAF causes significant outcomes at the lowest concentrations within 24 h of exposure [81].

BPA and its analogues disrupt spermatogenesis, leading to the deterioration of its outcome, which is proven by a reduction in sperm quality. Low sperm production induced by toxicants is usually associated with oxidative stress and the reduction of testosterone in the blood circulation [49,106]. Furthermore, the reduction in sperm development may also be due to abnormal SC causing insufficient nutrition, which is necessary for spermatogenesis [105]. According to previous studies, adult male rats’ exposure to BPA causes a decrease in sperm quality, proven by a reduction in sperm production, count, motility, viability, and the integrity of sperm acrosome and plasma membrane mitochondrial activity [64,65,66,67,75,76,77]. BPA at a dose of 50 mg/kg/bw for 14 days caused mild oedema in the LC, leading to a reduction in testosterone, thus lowering the sperm quality of adult male Wistar rats [77]. There is also an association reported between mitochondrial activity and motility in sperm because the mitochondria is the only source of ATP that enables the energy production necessary for sperm movement [76]. The sperm-specific ion calcium (Ca^2+^) channel (CatSper) is also crucial for sperm motility, hyperactivation, and acrosome reaction. This pH-sensitive channel is responsible for providing enough Ca^2+^ for sperm function [107]. Progesterone is a factor that influences the activation of the CatSper channel for sperm hyperactivation and acrosomal reaction to penetrate the oocyte [107]. Previous findings reported that the expression of the CatSper channel and charges were significantly downregulated and decreased after exposure to 10, 50, and 250 µg/kg/kg doses of BPA to the sperm mice orally [84]. These reductions parallel the finding where the motility and acrosome reaction in the presence of progesterone were significantly decreased as well. Exposure of healthy human sperm to 10 μM BPA analogues showed a similar effect on the CatSper channel’s ability. In a study, the scholars found that BPG, BPAF, BPBP, BPC, and BPB are potent chemicals that inhibit progesterone-induced Ca^2+^ [85]. These BPA analogues are shown to affect Ca^2+^ signaling, which can interfere with normal CatSper signaling and result in infertility [85]. Table 3 shows the effects of BPA and its analogues on spermatogenesis in the male reproductive system.

## 5. The Effects of BPA and Its Analogues on Male Reproductive Hormones: Human Biological Studies Evidence

Human epidemiology findings show a strengthened impact of BPA on the male reproductive system, involving the sexual hormones and sperm characteristics, through in vivo and in vitro studies. However, to the best of our knowledge, the number of human studies on the effects of BPA analogues on the male reproductive system focusing on the HPG axis is still limited. Most human studies have shown an association between the presence of BPA and its analogues in biological samples, such as urine and serum, with male sexual hormones and its effects on spermatogenesis outcomes, such as sperm characteristics parameters. A cross-sectional study was carried out between male children and adolescents in the United States of America. This study found a significant association between a high concentration of BPA in the urine and a low level of total testosterone in the serum of male adolescents only [108]. Researchers have estimated that 1 unit of BPA might lower the total testosterone levels by approximately 50% [108]. The finding prevailed for associations between BPA and other male reproductive hormones, such as AD and FSH, in men who work in the epoxy resin industry in Shanghai, China [109]. Liu et al. [109] found that increased BPA concentration in the urine indicated a significant decrease in AD and FSH levels in the worker’s serum [109]. Another study among men in the same industry in Guangdong, China, also presented the same findings [110]. The researcher found that BPA concentration and the duration of BPA exposure also influence hormone levels. Higher concentration and longer duration of BPA exposure decreased AD levels increasingly. Furthermore, Zhuang et al. [110] also found that a high concentration and a longer duration of BPA exposure also increased the sex hormone binding globulin (SHBG) in the serum of workers in the industry [110]. SHBG is a globulin protein that carries testosterone, dihydrotestosterone (DHT), and E2. Among these hormones, testosterone is carried the most by this protein. Therefore, an increase in SHBG level suggests that the testosterone level has decreased in the serum of the workers exposed to BPA. However, when comparing worker and non-worker groups, no association was found on the levels of SHBG, total testosterone, inhibin B, and AD [110].

Meanwhile, a study by Lassen et al. [111] found that increased BPA concentration in the urine significantly increased the total and free testosterone, LH, and E2 levels in the serum of the population of young men in Denmark [111]. Furthermore, a cross-sectional study conducted by Adoamnei et al. [112] found that increased concentrations of BPA in the urine also present a significant increase in the LH level among the population of young men in Spain. However, no association was found between BPA and FSH, free testosterone, SHBG, inhibin B, or E2 levels in the same population [112]. BPA acts as an anti-androgen agent by its ability to competitively bind to the AR, causing an increase in testosterone levels in circulation. However, even though BPA can bind to AR, it does not have any effects on testosterone, as a massive amount of BPA is required in serum to exhibit any effect as an antagonist towards AR. Therefore, the HPG axis is being induced to secrete LH for steroidogenesis activation in the LC, leading to increasing testosterone formation. This mechanism explains the increased levels of LH and free testosterone reported by Lessen et al. [111] and Adoamnei et al. [112].

BPA is known to raise oestrogenic activity either by stimulating or inhibiting the ER [113]. BPA has been reported to have a higher affinity towards ERβ than ERα in an in vitro study [112]. The action of BPA towards these receptors appears to be very complex, but the response depends on the presence of the ER subtypes (ERα and ERβ) and the co-regulatory protein, which either acts as a co-activator protein (stimulate) or the co-repressor protein (inhibit) [113]. The stimulation or inhibition of ER causes an increase or decrease of E2 in circulation, respectively [112]. Several studies have reported increasing E2 levels in the blood after exposure to BPA [108,111]. BPA can strongly bind to oestrogen-related receptors, such as ERR-γ, which interfere with the steroid synthetase functions and gene expression involved in steroidogenesis [110]. BPA has also been identified to bind with the ER at the anterior and posterior pituitary gland, leading to HPG axis disturbance and activation of prolactin secretion. The increased prolactin level in men who were occupationally exposed to BPA was evidence for this [109]. Furthermore, BPA can also increase the E2 level by its ability to activate an alternative steroidogenesis pathway. In humans, the Δ5 pathway is dominant; however, BPA may activate the alternative pathway where DHEA is converted into AD, which has a high incidence of stimulating the overproduction of E2 [109,110].

A retrospective study known as the ELEMENT project was carried out by Ferguson et al. [114], who reported no association between BPA levels found in mothers’ urine during pregnancy with SHBG, inhibin B, or free and total testosterone levels in the serum of male children aged between 8 and 14 years in Mexico [114]. Furthermore, the same study also found no association between BPA in the urine of the male children with SHBG, inhibin B, and the free and total testosterone levels in the serum [114]. However, a retrospective study done by Hart et al. [115] found a significant weak positive correlation between BPA in the serum of pregnant women and sperm motility and concentration in the semen of young men aged between 20 and 22 years old in Australia [115]. BPA was found in almost 90% of the maternal serum sample [115]. Furthermore, other epidemiological studies showed that a high concentration of BPA decreased sperm quality, proven by decreasing sperm concentrations, count, and motility [111,112,116,117]. A study by Lessen et al. [111] found that a high concentration of BPA present in the urine caused a decrease in the percentage of progressively motile sperm in the semen of young men in Denmark [111]. The same finding was reported between BPA and sperm count and concentration in young men aged 18–23 years in Spain. The high BPA concentration found in the urine of young men decreased the sperm count and concentration. However, the sperm motility and morphology in the same study population did not show any alterations [112]. Furthermore, there was no association between BPA and semen analysis, such as sperm morphology, sperm concentration, total sperm count, and semen volume, among fertile men aged over 18 years in Michigan and Texas [118]. Interestingly, BPA has also been identified to affect semen quality in IVF patients in Slovenia, where sperm count, concentration, vitality and motility decreased with the increasing concentration of BPA in the urine [116].

BPA analogues, such as BPS, also presented adverse effects in semen analysis among the population of young men involved in the FEPOS project in Denmark. The semen volume was lowered in subjects who had high BPA in their urine [119]. In contrast, Abou Ghayda et al. [117] found that infertile patients in Boston, MA, USA, with a high BPS concentration showed a high semen volume; however, this semen has a lower sperm concentration [117]. Low sperm concentration was found in infertile patients who were exposed to BPS. The researcher also reported that an increased level of BPS causes a significant decrease in sperm quality among obese or overweight patients who have a BMI >25 kg/m^2^. Therefore, BPS can increase the severity of sperm defects observed in obese or overweight infertile patients. Table 4 summarises human epidemiological studies of BPA and its analogues towards the male reproductive system.

## 6. Discussion

There is significant evidence that endocrine disrupting chemicals such as BPA and its analogues are an additional risk factor to be considered. Understanding the link between them and hormonal physiological function in the male reproductive system would aid in ensuring that suitable efforts are made to enhance public awareness and prevent their detrimental impacts on health.

BPA and its analogues have been the topic of extensive research and controversy in recent decades, and the amount of information available about them today is astounding. There are numerous papers that dealing with BPA and its analogues on human health, and the trend is for more to be published in the future. It is considered that the effects of BPA and its analogues on human health are a relatively new field with many unresolved problems. As previously stated, BPA and its analogues are commonly used substances in developing and underdeveloped countries. This means that scientists cannot dismiss the possibility that one of the functions of these compounds is to disturb the endocrine system. Numerous studies, both epidemiological and experimental, published in recent years have contributed to our understanding of some of the features and how they influence the hormonal physiological functions in the male reproductive system.

BPA and its analogues have a negative impact on hormonal physiological functions in the male reproductive system, which have been proven in vitro [55,78,79], in animals [56,64,65,66,67,68,69,70,72,74,75,76,77,80] and in human studies [108,109,110,111,116,118]. These hormonal physiological dysfunctions affect spermatogenesis outcomes. Some studies have shown decreased LH, FSH and testosterone levels when exposed to certain concentrations of BPA and its analogues as occurs in the HPG axis, specifically when this exposure occurs during adulthood [64,65,66,67,68,75,76,77]. The result of this review suggests that BPA and its analogues are capable of competitively binding to AR and ER and stimulating KiSS1 expression in the hypothalamus of the brain, causing the HPG axis feedback mechanism disturbance. These effects will alter GnRH secretion, resulting in variations in pituitary secretion of LH and FSH, with most studies reporting lower levels of these hormones. It is, therefore, logical that experimental animal studies showed low levels of testosterone as the negative feedback mechanism for these phenomena. Furthermore, BPA and 17-beta-oestrogen molecules are structurally similar; therefore, the former can adhere to the ER and mimic the oestrogenic effects in a human study [110,111], which is supported by the animal data [60,72,73]. Epidemiology studies have also shown that BPA and its analogues found in human urine are associated with the disturbance of male reproductive hormones via its effects as anti-androgenic and anti-oestrogenic agents [110,111,112,116].

Not only involving the HPG axis mechanism, BPA and its analogues could interfere with the steroidogenesis pathway via upregulation and downregulation of genes and proteins such as CYP and HSD, causing steroidogenesis disturbance [110,112]. BPA and its analogues can activate an alternative pathway known as the aromatase pathway, which alternates from testosterone synthesis to oestrogen synthesis [110,112]. This aromatase activity happens due to the upregulation of genes and protein expression of CYP19A1, which has been proven in in vitro and animal studies [56,59,74]. One of the primary results of this analysis is that BPA and its analogues have the potential to alter the steroidogenesis pathway, resulting in outcomes that are analogous to those of prostate cancer, such as high oestrogen levels in blood circulation. Therefore, BPA and its analogues exposure could be listed as one of the risk factors for prostate cancer.

In the Section 3 of the findings of this literature review, we examined articles that investigated the effects of BPA and its analogues on spermatogenesis outcomes. The disturbance of male reproductive hormones reduces spermatogenesis outcomes proven by sperm and semen quality defects. Testosterone and FSH play important roles in spermatogenesis; therefore, a decrease in these hormones will result in sperm quality defects, which were reported in animals and cell cultures exposed to BPA and its analogues [64,65,66,67,68,69,70,75,76,77,80,81,82]. The human findings also reported a decrease in sperm and semen quality when exposed to BPA and its analogues [111,112,116]. CatSper channel is another factor that influences sperm motility, hyperactivation and acrosomal reaction to penetrate the oocyte. As discussed previously, CatSper is one of the channels found in the sperm responsible for the influx of Ca^2+^ for sperm function. Therefore, downregulation and alteration in this channel after BPA and its analogues exposure could lead to immotile sperm and reduction in oocyte penetration ability even with the presence of progesterone. All these events in spermatogenesis disturbances lead to sperm quality defects resulting in infertility [85].

The significant drawback of this work is that most publications examine animal studies rather than human studies, owing to the logical and ethical challenges of doing this type of study in humans. Some findings are parallel between humans and animals; however, some findings showed differences, such as the doses used in animal and human studies. The low observed adverse effects level (LOAEL) dose for BPA in a mammalian animal is 50 mg/kg/bw. Even though the dose of BPA used in the previous studies was below the LOAEL, it reportedly caused changes in the reproductive hormone, sperm characteristic, and histological of the testis in laboratory animals [66,70,71,72,76]. Rats were more susceptible to BPA when compared to humans. Therefore, the adverse effect is more severe in rats, specifically the male reproductive system, proven by the disturbance of reproductive hormone, gene, and protein expression in steroidogenesis and reduced sperm quality.

Even though the BPA concentration found in human urine is lower than the tolerable daily intake (TDI), which is below than 4 µg/kg, significant negative effects were found in our literature findings proven by alterations in the reproductive hormones, sperm characteristics, and semen quality. However, the data from human biomonitoring studies are uncertain, which might be due to different metabolism rates in individuals influencing their ability to excrete the BPA [9]. Furthermore, the concentration of BPA and the duration of exposure might also be significant factors that must be considered in assessing the effect of BPA on the hormones’ physiological function in the male reproductive system. There are a few possible challenges from the previous literature findings which derive interpretation concern: subject population (fertile versus infertile, young adult versus children; BPA and its analogues exposure levels (due to non-linear effects of BPA); sampling frequency limitations (sample may not be representative of exposure such as retrospective study); and BPA exposure window which may not be related to the spermatogenesis outcomes because the spermatogenesis process in the human takes about three months to be complete [9]. Another problem is that the effects of BPA and its analogues on physiological hormonal functions were explored without taking into account probable interactions with other environmental factors.

**Figure 1:**
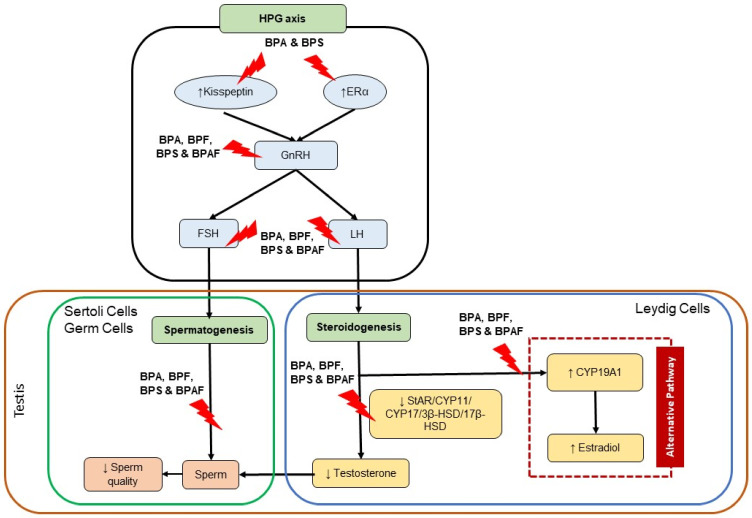
Possible mechanism of BPA and its analogues on the hormonal physiology of male reproductive system. Abbreviations: ↓ inhibit/decrease, ↑ enhance/increase.

Figure 1 shows the possible mechanism of BPA and its analogues on the hormonal physiology pathway of the male reproductive system. BPA and its analogues negatively impact the physiological hormonal functions in the male reproductive system and its outcome, which were proven by sperm and semen quality defects. The BPA and its analogues can alter the HPG axis by increasing the expression of KiSS1 and competitively bind with ERα in the brain’s hypothalamus. These effects will influence GnRH secretion, leading to changes in the pituitary secretion of LH and FSH, which most of the studies reported decreased levels of these hormones. Decreased LH levels in the plasma could disturb the steroidogenesis process in Leydig cells, resulting in decreased testosterone levels. Furthermore, these endocrine disrupting chemicals were also reported to alter the StAR protein, HSD and CYP450 genes and enzymes in the mitochondrial and ER of Leydig cells involved in the steroidogenesis pathway. These alterations also lead to the decrease of testosterone synthesis. Therefore, once testosterone synthesis is inhibited, the alternative pathway will be activated. BPA and its analogues can activate this alternative pathway of steroidogenesis by activating aromatase activity, thus increasing the formation of E2. As the consequences of HPG axis disturbance and activation of the alternative steroidogenesis pathway specifically proven by the FSH and testosterone levels decreased, respectively, these phenomena lead to the disturbance of spermatogenesis reported in most studies. Therefore, the spermatogenesis outcome, such as sperm quality, will have deteriorated.

## 7. Conclusions

In conclusion, BPA and its analogues may be an additional risk factor to consider because they disrupt the hormonal physiological functions of the male reproductive system. The results of the experimental studies mostly point to BPA and its analogues having the ability to disrupt the endocrine system, resulting in HPG axis disturbances and activation of the steroidogenesis alternative pathway, which is why more experimental and epidemiological research will be required to establish the scale of the effects caused by these chemicals in large populations and its molecular mechanism to a better understanding of the connection between HPG axis, steroidogenesis, and spermatogenesis outcomes. Despite the fact that many nations have adopted policies to limit exposure to BPA and its analogues in their populations, epidemiological research on humans imply that the same abnormalities seen in experimental studies on animals may be detected. Understanding the association between BPA and its analogues and physiological hormonal activities can aid in raising public awareness and implementing public health campaigns to prevent exposure to these compounds, particularly among those attempting to conceive and elderly populations.

## Figures and Tables

**Table 1:** The effects of BPA and its analogues on the hypothalamus-pituitary-gonadal axis in male reproductive system.

Type of Bisphenol	Purity (Manufacturer)	Dose (Route)	Animal	Duration of Exposure	Findings	Author
BPA	99% (Sigma-Aldrich, St. Louis, MO, USA)	50 µg/kg/bw (drinking water)	Adult male Wistar rats	Perinatal exposure (From gestation day 9 until lactation day 20)	↑ Kiss1 mRNA expression (hypothalamus) ↓ ERα, ERβ (hypothalamus) ↑ testosterone (blood) ↓ estradiol (blood)	[71]
BPA	>99% (Sigma-Aldrich)	2 µg/kg/bw (s.c)	Offspring male SD	Perinatal exposure Day 10 of gestation until day 7 of lactation	↑ Kiss1 mRNA expression (brain) ↑ GnRH neuron ↑ LH, estradiol (blood) ↓ testosterone (blood)	[72]
BPA	-	50 mg/kg/bw (s.c.)	Male pup Long Evans rats	2 days Postnatal day 0–2	↑ expression of Kiss1 (brain) ↑ expression of ERα (brain)	[73]
BPA	MERCK, Kenilworth, NJ, USA	50 mg/kg/bw (i.p.)	Adult male SD rats	Alternate day until 21 days	↓ FSH, LH, testosterone (serum) ↑ estradiol (serum)	[75]
BPA	Sigma-Aldrich	5 or 25 mg/kg/bw (oral gavage)	Adult male Wistar rats	40 days	↑ expression of Gnrhr, Lhb, Fshb, ERβ, AR mRNA (pituitary) ↓ Gnrh, ERα (hypothalamus) ↓ FSH, LH, testosterone (blood) ↑ estradiol (blood)	[76]
BPA	>99% (Sigma-Aldrich)	50 mg/kg/bw (oral gavage)	Adult male Wistar rats	14 days	↓ FSH, LH, testosterone (serum)	[77]
BPA	Sigma-Aldrich	50 mg/kg/bw (oral gavage)	Adult male Wistar rats	30 days	↓ FSH, LH, testosterone, E2 (plasma)	[64]
BPA	Sigma-Aldrich	200 mg/kg (oral gavage)	Adult male SD rats	42 days	↓ FSH, LH, testosterone (blood)	[65]
BPA	-	25 mg/kg/bw (i.p.)	Adult male SD rats	Alternate day for 30 days	↓ FSH, LH, testosterone (plasma)	[66]
BPA	Gracia chengdu chemical technology co, Chengdu, Sichuan, China.	200 mg/kg (oral gavage)	Adult male SD rats	28 days	↓ FSH, LH, testosterone (blood)	[67]
BPF	99% purity (Santa Cruz Biotechnologie, Dallas, TX, USA)	1, 5, 25, 50, and 100 mg/kg/bw (Oral gavage)	Adult male SD rats	28 days	↓ FSH, LH, testosterone (plasma) ↓ testicular testosterone	[68]
BPA/BPS	>98%	1000 μg/L (BPA) 100 μg/L (BPS)	Transgenic zebrafish (embryo)	2 h of post fertilization until 25 or 20 h of post fertilization	BPA: ↑ number of GnRH3 neuron at 25 hpf (hypothalamus) ↑ expression of Kiss1 mRNA, Kiss1 receptor, gnrh3, lhβ, and fshβ after 120 h post fertilization (embryo) ↑ expression of ERα at 25 hpf BPS: ↑ number of GnRH3 neuron at 25 hpf (hypothalamus) ↑ expression of mRNA Kiss1 and gnrh3 at 25 hpf (hypothalamus) ↑ expression of ERα 25 hpf	[62]
BPF/BPS	99% purity (Santa Cruz Biotechnologie)	5, 50, and 500 mg/kg/bw (Oral gavage)	Adult male SD rats	28 days	BPF: ↓ testicular and plasma testosterone BPS: ↓ testicular and plasma testosterone	[69]
BPF	> 98% (J&K Scientific Ltd., Newark, DE, USA)	0.1 and 1 mg/L in aquarium water Renewed 50% of water every 2 days	Male Zebrafish	21 days	↑ expression of GnRH2, GnRH3, GnRHR1, and GnRHR2 (Brain) ↑ FSHR, LHR (testis) ↓ testosterone (testis) ↑ estradiol (testis)	[56]
BPS/BPF/ BPAF	98% (Sigma-Aldrich)	In vitro: BPAF: 0.076 µm BPA: 2.8 µm BPF: 10.6 µm	Zebrafish (embryo and larvae)	From day 1 of hdf until 7-dpf larva	BPAF, BPA, BPF: ↑ affinity toward binding of ERα (in vitro) BPS: No affinity toward the ERα receptor (in vitro)	[59]
BPS	-	1, 50 µg/kg/bw (oral gavage)	Adult male SD rats	28 days	↓ testicular and plasma testosterone	[70]
BPAF	99% (Sigma-Aldrich)	25 and 125 µg/L	Offspring male zebrafish (embryo)	120 days (exposure during embryo and larva stages)	↑ gnrh2, fshβ, lhβ, and cyp19b in 125 µg/L ↑ estradiol ↓ testosterone	[74]

Abbreviations: ↑ Increase; ↓ Decrease.

**Table 2:** The effects of BPA and its analogues on the steroidogenesis in male reproductive system.

Type of Bisphenol	Purity (Manufacturer)	Dose (Route)	Animal	Duration of Exposure	Findings	Author
BPA	99% (Sigma-Aldrich)	50 mg/kg/bw (oral gavage)	Adult male Wistar rats	14 days	↓ testosterone (serum)	[80]
BPA	99% (Sigma-Aldrich)	50 µg/kg/bw (drinking water)	Adult male Wistar rats	Perinatal exposure (gestation day 9 until lactation day 20)	↑ testosterone (blood) ↓ estradiol (blood)	[71]
BPA	>99% (Sigma-Aldrich)	2 µg/kg/bw (s.c)	Offspring male SD rats	Perinatal exposure Day 10 of gestation until day 7 of lactation	↑ estradiol (blood) ↓ testosterone (blood)	[72]
BPA	Sigma-Aldrich	5 or 25 mg/kg/bw (oral gavage)	Adult male Wistar rats	40 days	↓ testosterone (blood) ↑ estradiol (blood)	[76]
BPA	MERCKS	50 mg/kg/bw (i.p.)	Adult male SD rats	Alternate day until 21 days	↓ testosterone (serum) ↑ estradiol (serum)	[75]
BPA	Gracia chengdu chemical technology co.	200 mg/kg (oral gavage)	Adult male SD rats	28 days	↓ testosterone (blood) ↓ expression of mRNA StAR, CYP11A1, 3β-HSD, CYP17A1 (testis)	[67]
BPA	Sigma-Aldrich	200 mg/kg (oral gavage)	Adult male SD rats	42 days	↓ testosterone (blood) ↓ expression of protein StAR, CYP11A1, 17β-HSD, 3β-HSD, CYP17A1 (testis)	[65]
BPA	Sigma-Aldrich	50 mg/kg/bw (oral gavage)	Adult male Wistar rats	30 days	↓ testosterone, estradiol (plasma)	[64]
BPA	-	25 mg/kg/bw (i.p.)	Adult male SD rats	Alternate day of 30 days	↓ testosterone (plasma)	[66]
BPF	99% (Santa Cruz Biotechnologie)	1, 5, 25, 50, and 100 mg/kg/bw	Adult male SD rats	28 days	↓ testosterone (plasma) ↓ testosterone (testis)	[68]
BPF/BPS	99% (Santa Cruz Biotechnologie)	5, 50, and 500 mg/kg/bw	Adult male SD rats	28 days	BPF: ↓ testicular and plasma testosterone BPS: ↓ testicular and plasma testosterone	[69]
BPF	> 98% (J&K Scientific Ltd)	0.1 and 1 mg/L in aquarium water	Male Zebrafish	21 days	↓ testosterone (homogenate) ↑ estradiol (homogenate) ↑ expression of CYP19A1b (aromatase)(Brain) ↑ expression of mRNA CYP11A, CYP19A (testis) ↓ expression of mRNA StAR, CYP17, 17βHSD (testis)	[56]
BPS/BPF/ BPAF	98% (Sigma-Aldrich)	In vivo: 1 µm In vitro: BPAF: 0.076 µm BPA:2.8 µm BPF: 10.6 µm	Zebrafish (embryo and larvae)	From day 1 of hdf until 7-dpf larva	BPAF, BPF, BPS: ↑ expression of mRNA CYP19A1 gene in 7-dpf (embryo)	[59]
BPS	-	1, 50 µg/kg/bw (oral gavage)	Adult male SD rats	28 days	↓ testicular and plasma testosterone	[70]
BPAF	99% (Sigma–Aldrich)	25 and 125 µg/L	Offspring male zebrafish (embryo)	120 days through exposure of embryo and larva stages -	↑ estradiol ↓ testosterone ↑ CYP19b (brain) ↑ expression of mRNA CYP19A and CYP11A1 (testis) ↓ expression of mRNA StAR and CYP17 (testis)	[74]
BPF/BPS	BPF (>99%) BPS (>98%)	BPF: 0.01–100 µM BPS: 0.01–30 µM	MA-10 Leydig cell culture	48 h	BPF, BPS: ↑ testosterone secretion ↑ expression of 5αRed1	[55]
BPF/BPS	-	10, 100, 1000, 10,000 nmol/L	Mouse fetal testis assay (mFeTA)	1–3 days	BPF, BPS (10 000 nmol/L): ↓ testosterone secretion ↓ expression of mRNA StAR, HSD3β1 and CYP17A1	[78]
BPF/BPS/ BPAF	BPF (99%) BPS (98%) BPAF (99%)	0.1, 1, 10, 30, 50 and 70 µM	Human adrenocortical carcinoma cell line (H295R)	-	BPF: ↑ estradiol and progesterone secretion (dose-dependent manner) ↓ expression of mRNA HSD3β2 (50 µM) and CYP17A1 (dose-dependent manner) BPS: ↓ testosterone secretion (dose-dependent manner) ↓ expression of mRNA CYP17A1 BPAF: ↓ testosterone secretion (dose-dependent manner) ↑ progesterone secretion ↓ expression of mRNA CYP17A1, HSD3β2	[79]

Abbreviations: ↑ Increase; ↓ Decrease.

**Table 3:** The effects of BPA and its analogues on spermatogenesis in the male reproductive system.

Type of Bisphenol	Purity (Manufacturer)	Dose (Route)	Animal	Duration of Exposure	Findings	Author
BPA	MERCK	50 mg/kg/bw (i.p.)	Adult male SD rats	Alternate day until 21 days	Sperm: ↓ sperm count, motility, viability	[75]
BPA	Sigma-Aldrich	50 mg/kg/bw (Oral gavage)	Adult male SD rats	52 days	Histopathology: Vacuolated and degeneration of germ cells	[80]
BPA	Sigma-Aldrich	5 or 25 mg/kg/bw (Oral gavage)	Adult male Wistar rats	40 days	Sperm: ↓ total and daily sperm production, integrity of acrosome, plasma membrane and mitochondria activity in sperm	[76]
BPA	99% (Sigma-Aldrich)	50 mg/kg/bw	Adult male Wistar rats	14 days	Histopathology: Leydig cells mild edema Spermatocyte depletion Spermatogenesis from weak to arrest. Sperm: ↓ daily sperm production, sperm count, sperm motility	[77]
BPA	Sigma-Aldrich	50 mg/kg/bw (Oral gavage)	Adult male Wistar rats	30 days	Histopathology: ↓ diameter and epithelial height of seminiferous tubule Atrophy and separation of germinal epithelium Sperm: ↓ sperm count	[64]
BPA	Sigma-Aldrich	200 mg/kg (Oral gavage)	Adult male SD rats	42 days	Sperm: ↓ sperm count, daily sperm production, motility	[65]
BPA	-	25 mg/kg/bw (i.p.)	Adult male SD rats	Alternate day of 30 days	Histopathology: Degeneration and vacuolation of germ cells Sperm: ↓ sperm count, motility, and viability	[66]
BPA	Gracia chengdu chemical technology co.	200 mg/kg (Oral gavage)	Adult male SD rats	28 days	Histopathology: ↓ quantity of mature sperm, Longer spermatid Disorganization of germ cells Sperm: ↓ sperm count, motility	[67]
BPF	99% (Santa Cruz Biotechnologie)	1, 5, 25, 50, 100 mg/kg/bw	Adult male SD rats	28 days	Histopathology: ↓ germinal epithelial height Absence of sperm in lumen	[68]
BPF/BPS	99% (Santa Cruz Biotechnologie)	5, 50, 500 mg/kg/bw	Adult male SD rats	28 days	Histopathology: BPF: Seminiferous tubules irregular Longer spermatid BPS: Absence of sperm in lumen	[69]
BPS	99% (Santa Cruz Biotechnologie)	25, 50 µg/kg/bw (Oral gavage)	Adult male SD rats	28 days	Histopathology: ↓ epithelial of seminiferous tubules Spermatid become longer.	[70]
BPA/BPAF /BPS	BPA (>99%) BPS (98%) BPAF (98%)	25, 50, 100 µM	C18-4 spermatogonial cell line	24–72 h	BPA: ↓ the cell viability after 24 h (100 µM) ↑ DNA damage after 48 h (50 µM) BPAF: ↓ the cell viability after 24 h (50 µM) ↑ DNA damage after 24 h (25 µM) BPS: ↓ the cell viability after 24 h (100 µM) ↑ DNA damage after 24 h (50 µM)	[81]
BPA	Sigma-Aldrich	10 and 50 mg/kg/bw	Adult male wistar rat	-	↓ occludin (10 mg/kg for 11 weeks and 50 mg/kg for 4 weeks) ↓ nectin-3 (50 mg/kg for 2–4 weeks)	[105]
BPA	-	20 µM	Sertoli cells	-	↓ occludin (after 48 h) ↓ Z0-1 (after 6 and 48 h) ↓ cells viability after 6 and 48 h ↓ androgen receptor after 6 and 48 h	[82]
BPA	Sigma-Aldrich	25 and 100 µM	Sertoli cells isolated from 20 days of wistar rats	-	↓ occludin and ZO-1 (both doses) ↑ conexxin (both dosage) Significantly perturb the tight junction barrier at dosage 100 µM (*p* < 0.05)	[105]
BPA	Sigma-aldrich (US)	10, 50,250 µg/kg/D (Oral gavage)	Sperm of C57BL/6 mice	8 weeks	↓ sperm motility (*p* < 0.05) ↓ Progesterone-induced acrosome reaction (*p* < 0.05)	[84]
BPG, BPAF, BPC, BADGE, BPB	Sigma-aldrich (MO,US)	Ca^2+^ signal: 10 µM Progesterone-induced Ca^2+^ signal: BPF: 5 µM BPAF&BPBP: 10 µM BPC, BADGE, BPB: 50 µM	Healthy human semen	-	↑ Ca^2+^ signaling ↓ Progesterone-induced Ca^2+^ signal	[85]

Abbreviations: ↑ Increase; ↓ Decrease.

**Table 4:** The summary of human epidemiological studies of BPA and its analogues towards male reproductive system.

Type of Bispheno	Study Design	Study Population (Age) (Project Name)	Country (Sample Population)	Biological Sample	[Bisphenol] Detected in Biological Sample (Mean/ Median)	Findings	Beta Coefficient	Significant Values	Author
BPA	Cross sectional study	Male children (6–11 y.o) Male Adolescents (12–19 y.o) (NHANES Project)	USA (*n* = 588)	Urine Serum	Mean: male children 1.74 ng/mL (urine) Mean: male adolescents 1.94 ng/mL (urine)	Reproductive hormones: No association between BPA and reproductive hormones in male children across the quartiles. Increased BPA level caused a significant decrease in TT in male adolescents across the quartiles.	Q2: β = −49.34% Q3: β = −36.87% Q4: β = −53.70%	*p* < 0.05	[108]
BPA	Cross sectional study	Male worker of epoxy resin manufacturer (16–63 y.o)	Shanghai, China (*n* = 592)	Urine Serum	Median occupational exposure: 685.9 µg/g Cr (urine) Median non-occupational exposure: 4.2 µg/gCr (urine)	Reproductive hormones: Increased level of BPA cause significant increase in: Prolactin SHBG E2 across the quartiles Increased level of BPA cause significant decreased in levels: AD FSH across the quartiles			[109]
							β = 0.0589 ng/mL	*p* < 0.001	
							β = 0.0293 nmol/L	*p* < 0.01	
							β = 0.0362 pg/mL	*p* < 0.001	
	
							β = −0.0367 ng/mL	*p* < 0.001	
							β = −0.024 mIU/mL	*p* < 0.05	
	
BPA	Cross sectional study	Male worker of epoxy resin manu-facturer	Guang-dong, China (*n* = 559)	Serum	Median workers: 8.75 ng/mL (serum) Median non-workers: 3.37 ng/mL (serum)	Reproductive hormones: No association between workers and non-workers on the level of SHBG, TT, INB and AD			[110]
						Increased exposure time caused significant decreased in median AD level among workers.	-	*p* < 0.001	
						Increased exposure time caused significant increase in median SHBG level among workers.	-	*p* < 0.05	
						Increased BPA level caused significant increase in median SHBG level among workers.	β = 2.79 nmol/L	*p* < 0.05	
						Increased of BPA level caused significant decreased in median AD level among workers.	β = −0.18 ng/mL	*p* < 0.001	
BPA	Cross sectional study	Young men	Denmark (*n* = 308)	Urine Serum Semen	Median: 3.74 ng/mL (Osm)(urine)	Reproductive hormones: Increased level of BPA caused significant increase in: TT FT LH E2 across the quartiles.			[111]
							β = 0.7 nmol/L	*p* < 0.01	
							β = 2.7%	*p* < 0.05	
							β = 3.5%	*p* < 0.05	
							β = 2.7%	*p* < 0.05	
	
						Sperm characteristics: Increased level of BPA caused a significant decreased in percentage of progressive motile spermatozoa across the quartiles.	β = −1.82%	*p* < 0.01	
BPA	Cross sectional study	Young men (18–23 y.o)	Spain (*n* = 215)	Urine Serum Semen	Mean: 1.8 µg/g (urine)	Reproductive hormones: Increased level of BPA caused significant increase in LH level across the quartiles. No association between BPA and FSH, FT, SHBG, INB and E2 across the quartile.	β = 0.07 IU/L	*p* < 0.01	[112]
						Sperm characteristics: Increased level of BPA caused significant decreased in sperm characteristic across the quartiles: concentration sperm count No association between BPA and sperm motility and morphology			
							β = −0.04 Mill./mL	*p* < 0.01	
							β = −0.05 Mill.	*p* < 0.01	
	
BPA	Retro-spective cohort	Pregnant woman Male chil-dren (8–14 y.o) (ELEMENT project)	Mexico (*n* = 118)	Urine Urine Serum	Mean: 0.7 ng/mL (urine) Mean: 1.1 ng/mL (urine)	Reproductive hormones: No association between prenatal urinary BPA and the boy sex hormones in the level of SHBG, INB, TT, E2, DHEAS and FT	-	-	[114]
						No association between child-hood urinary BPA and the boy sex hormones in the level of SHBG, INB, TT, E2, DHEAS and FT	-	-	
BPA	Retro-spective cohort	Pregnant woman (Week 18 & 34) Young men (20–22 y.o)	Australia (*n* = 423)	Serum (mother) Semen	Median: 0.25 µg/L (serum)	Sperm characteristics: Maternal exposure of BPA caused significant changes in sperm characteristics of young men such as increased in the sperm concentration and motility.	-	*p* < 0.05	[115]
BPA	Pro-spective cohort	Men IVF patient (34.05 y.o)	Slovenia (*n* = 149)	Semen	Mean: 1.33 ng/mg (urine)	Sperm characteristics: Increase concentration of BPA cause significant decrease in: sperm concentration sperm count sperm motility sperm vitality			[116]
	
	
							β = −0.219, R2 = 0.071	*p* = 0.047	
							β = −0.241, R2 = 0.092	*p* = 0.039	
							β = −0.273, R2 = 0.075	*p* = 0.043	
							β = −0.266, R2 = 0.052	*p* = 0.026	
BPA	Pro-spective cohort	Fertile men (>18 y.o)	Michigan and Texas (*n* = 418)	Urine Semen	Mean: 0.51 µg/g (urine)	Sperm characteristics: Increased level of BPA caused significant decreased in sperm DNA fragmentation. No association between BPA and semen analysis (sperm morphology, sperm concentration, total sperm count, semen volume).	β = −0.0544	*p* < 0.05	[118]
BPA/ BPF/ BPS	Cross sectional study	Young men (18–20 y.o) FEPOS	Denmark (*n* = 556)	Urine Semen	BPA (urine) Q1: <0.68 ng/mL Q3: 1.3–2.74 ng/mL BPF (urine) Q1: <0.06 ng/mL Q3: 0.14–0.34 ng/mL BPS Q1: <0.03 ng/mL Q3: 0.06–0.17 ng/mL (urine)	Sperm characteristics: Percentage of motile spermatozoa in Q3 is significantly higher compared to Q1 in BPA and BPF exposures. Volume of semen per ejaculate in Q3 is significantly lower compared to Q1 in BPA and BPS exposures. No association between (BPA, BPF, and BPS) with the other semen analysis (sperm concentration, total sperm count, normal sperm morphology, motility and ejaculate volume).	β = 1.07% β = −0.87 mL	*p* < 0.05 *p* < 0.05	[119]
BPS	Cross sectional study	Infertile patient (18–56 y.o)	Boston, MA, USA (*n* = 158)	Urine Semen	Mean BPA: 0.77 µg/L (urine) Mean BPS: 0.37 µg/L (urine)	Semen characteristics: Volume of semen per ejaculate in Q2 is significantly higher compared to Q1 in BPS exposure. Sperm concentration in Q3 is significantly lower compared to Q1 in BPS exposure. Increased level of BPS caused significant decreased in sperm quality among obese/overweight men (BMI >25 kg/m^2^): sperm concentration total sperm count total motility	β = 3.0 mL β = −29.2 mil/mL	*p* < 0.05 *p* < 0.05	[117]

Abbreviations: ↑: Increase; ↓ Decrease

## Abbreviations

3β-HSD	3β-hydroxysteroid dehydrogenase
5αRed1	5α- reductase type 1
17β-HSD	17β-hydroxysteroid dehydrogenase
AA	arachidonic acid
AD	androstenedione
AR	androgen receptor
ATP	adenosine 5’-triphosphate
BPA	bisphenol A
BPAF	bisphenol AF
BPF	bisphenol F
BPS	bisphenol S
BTB	blood-testis-barrier
cAMP	cyclic adenosine monophosphate
CatSper	sperm-specific ion calcium (Ca^2+^) channel
CYP11A1	cytochrome P450 isoform 1A1
CYP17	cytochrome P450 isoform 17
CYP19A1	cytochrome P450 isoform 19A1
COX-2	cyclooxygenase-2
DHEA	dehydroepiandrosterone
E2	estradiol
EDCs	endocrine disrupting chemicals
EPA	Environmental Protection Agency
ER	estrogen receptor
ER α/β	estrogen receptor α/β
ERR-γ	estrogen-related receptor gamma
FSH	follicle-stimulating hormone
FSHβ	follicle-stimulating hormone beta
FT	free testosterone
GnRH	gonadotropin-releasing hormone
Gnrh2	gonadotropin-releasing hormone 2
GnRH3	gonadotropin-releasing hormone 3
GnRHR	GnRH receptor
GnRHR 1/2	GnRH receptor 1/2
GPR54	G-protein coupled receptor 54
HPG	hypothalamic–pituitary–gonadal axis
HSD	hydroxysteroid dehydrogenase
INB	inhibin B
IVF	in vitro fertilization
LC	Leydig cells
LH	luteinizing hormone
LHβ	luteinizing hormone beta
LHR	luteinizing hormone receptor
MAPK	mitogen-activated protein kinase
mFeTA	mouse fetal testicular cell assay
mRNA	messenger RNA
PGE-2	prostaglandin E2
PKA	protein kinase A
PSA	prostate-specific antigen
Q	quartile
SC	Sertoli cell
SHBG	Sex hormone binding globulin
StAR	Steroidogenic acute regulatory
TT	total testosterone
YO	years old
ZO-1	zona occludens-1
